# Interaction between environment, nutrient-derived metabolites and immunity: A possible role in malaria susceptibility/resistance in Fulani and Dogon of Mali

**DOI:** 10.1371/journal.pone.0189724

**Published:** 2017-12-20

**Authors:** Karim Traore, Mahamadou A Thera, Anne-Lise Bienvenu, Charles Arama, Guillaume Bonnot, Adeline Lavoignat, Ogobara K Doumbo, Stephane Picot

**Affiliations:** 1 Malaria Research and Training Centre, Université des Sciences, des Techniques et des Technologies de Bamako, MRTC/USTTB, Bamako, Mali; 2 Univ Lyon, Université Claude Bernard Lyon 1, Institut de Chimie et Biochimie Moléculaire et Supramoléculaire, UMR-5246 CNRS-INSA-CPE, Malaria Research Unit, Lyon, France; 3 Institut de Parasitologie et Mycologie Médicale, Hospices Civils de Lyon, Lyon, France; Baker IDI Heart and Diabetes Institute, AUSTRALIA

## Abstract

The role of some nutrient-derived metabolites on the innate and adaptive immune responses is now established. Global research approach investigating the interplay between environment, lifestyle and the host’s immune responses is crucial in the understanding of malaria susceptibility. Advanced Glycation end products (AGE), which are food-derived metabolites result from the link between reducing sugar and amino group of proteins, lipids or nucleic acids. The level of exposure to AGEs varies depending on the type of diet. The dysfunction of the immune system induced by AGE and the cellular receptors for AGEs (RAGE) in susceptibility to bacterial infection has been described. But no study has yet explored their role in susceptibility to malaria. Therefore, we aimed to evaluate systemic AGE and RAGE gene polymorphism in two sympatric populations with previously described difference of susceptibility to malaria. We measured by ELISA the plasma levels of AGEs, and their soluble receptors (sRAGE) from 170 volunteers (68 Fulani and 102 Dogon). We also determined by real-time quantitative PCR the expression of RAGE, and the -374 T/A, -429 T/C polymorphisms and 63 bp deletion by fragment length restriction polymorphism. The prevalence rate of *Plasmodium* in Fulani and Dogon were respectively 42.64% and 51.30% for *P*. *falciparum*, 5.88% and 6.5% for *P*. *malariae*, 0% and 2.6% for *P*. *ovale*. The average AGE was 12.65 μg/ml, and 496.48pg/ml for sRAGE. Highest levels of sRAGE were observed in Fulani (563,07pg/ml, 95% CI [547.81–580.13] vs 465.68pg/ml, 95% CI [331.19–467.51]) for Dogon, p = 0.00001. Fulani had the lowest mean of AGE (10.21μg/ml, 95% CI [8.02–10.92]) compared to Dogon (16.88μg/ml, 95% CI [13.92–17.96]; p = 0.00001. RAGE was more expressed in Dogon than Fulani (0.08 vs 0.04), P = 0.08. The -374A polymorphism vas more frequent in Fulani (32%) compared to Dogon (20%). The chronic exposure to dietary AGE could lead to immune responses impairment and polymorphism with implications in malaria susceptibility. More studies are necessary to better investigate this hypothesis.

## 1. Background

The role of diet-derived metabolites on the innate immune system as well as on the adaptive immune response has been established [1,2]. Recent papers published have documented the evident role of diet-derived metabolites in the development of lymphoid organs and the modulation of immune system [3], highlighting the interplay between the immune system and the external environment, specifically the diet. The presence of receptors for the nutrients-derivatives on the immune cells support strongly this interplay between the environment and immune system modulation [1,3,4].

The clinical outcome of the current episode and the susceptibility to the subsequent episodes are determined by the modulation of the immune response induced by the current malaria episode [5,6]. One of the new research areas of great interest on malaria could be the global approach combining both the environmental factors, host lifestyle, gut microbiota and immune system modulation. Investigating the interaction between these environmental, anthropological and biological factors could contribute in the understanding of malaria susceptibility. There are several data supporting the role of the gut microbiota and some nutrient-derived metabolites in the development and the modulation of immune system, and the susceptibility to malaria [3,7]. But there are lack of data on the role of metabolism in the susceptibility to malaria.

Several studies conducted in sympatric Fulani and Dogon from Mali and Fulani and Mossi from Burkina Faso have described differences of susceptibility to malaria [8–10]. Data available from these populations do not provide sufficient information to well elucidate the role of biological and environmental factors in the mechanisms underlying this difference of susceptibility [11–17]. These two ethnic groups live in the same malaria endemic areas. One of the main differences between these populations is the life style. There is evidence that the nutritional status and the metabolism of the host are critical in the clinical outcome of malaria infection [18–22] due to the modulation of the immune responses and oxidative stress [23,24].

Data published have documented the implication of diet-derived advanced glycation endproducts (AGEs) in the pathogenesis of chronic and inflammatory diseases [25,26] and the susceptibility to bacterial infections [27,28]. Recent studies have described the role of dietary AGE in the modulation of innate and adaptive immune responses as consequences of the interaction of AGEs with their specific cell receptors (Receptors for AGE: RAGE). The RAGEs are present on the immune cells and constitute a direct interplay between diet and immune system cells [1]. Despite the documented implication of AGE-RAGE axis signaling way in the modulation of immune responses types Th1/Th2 balance leading to chronic Th1response and oxidative stress, there are lack of data on the role of AGE in malaria susceptibility.

Dietary AGEs are the AGEs present in food. The quantity of AGEs in food depends on the cooking method and it increases proportionally with the temperature and the time of cocking. Populations who have well-cooked food as main feeding lifestyle (most of African population) are more exposed to AGE than those who have less or none cooked food (Fulani ethnic group in west Africa have milk and cuscus as main food). A possible role of AGE-RAGE interaction and RAGE gene polymorphism has been hypothesized in the difference of susceptibility to malaria between Fulani and Dogon [29].

This study aimed to evaluate the relation between malaria, AGE, RAGE expression and RAGE gene polymorphism in two sympatric populations with known difference of susceptibility to malaria in Mali.

## 2. Methods

### 2.1. Data collection

Data were collected from healthy participants during two cross-sectional surveys in July and September 2011. These data included PBMC, plasma and sociodemographic data. Individuals of all age groups were included after obtaining their informed consent. Participant were selected from two ethic groups based on voluntarism. The selection of these two ethnic groups as study population is based on previous data published describing differences of susceptibility to malaria in these ethnic groups [8–10]. As this was a pilot study to determine baseline data on AGE and RAGE in this population, a minimal sample size was not calculated.

### 2.1. Ethical clearance and subjects

The study protocol was approved by the ethic committee of Mali ‘‘Comte d’ethique de la FMPOS, approval letter reference: No 2011-59-/FMPOS”. All the participants have signed informed consent forms, for the minors, the parent of legal tutor signed the consent after their assent have been obtained. Samples were collected by cross-sectional surveys in the Fulani and Dogon ethnic groups of Mali from July to September 2011. In total 222 individuals (68 Fulani and 154 Dogon) were included in this study.

### 2.2. Determination of molecular prevalence of Plasmodium species by qPCR

Prevalence of *Plasmodium falciparum*, *malariae*, *ovale* and *vivax* was determined by quantitative real time PCR using the following specifics primers: 5’AGTGTGTATCAATCGAGTTTTC3’ (forward), 5’AGTTCCCCTAGAATAGTTACA3’ (revers) for *P*. *falciparum*, 5’ATCTCTTTTGCTATTTTTTAGTATTGGAGA3’ (forward), 5’CCAAAGACTTTGATTTCTCAT3’ (revers) for *P*. *ovale*, 5’ATAACATAGTTGTACGTTAAGAATAACCGC3’ (forward), 5’AAAATTCCCATGCATAAAAAATTATACAAA3’ (revers) for *P*. *malariae;* 5’CGCTTCTAGCTTAATCCACATAACTGATAC3’ (forward), 5’ACTTCCAAGCCGAAGCAAAGAAAGTCCTTA 3’ (revers) for *P*. *vivax*.

### 2.3. Determination of plasma AGE by ELISA

OxiSelect™ Advanced Glycation End Product (AGE) Competitive ELISA Kit (STA-817-5) was used according the manufacturer’s instructions. Briefly, the Plate was coated with 100 μl of AGE conjugate in each well and incubated over night at 4°C. The plate is then washed twice with the 1X PBS and blocked with 200 μl of the assay diluent in each well and stored at 4° until use. A standard is prepared with serial dilutions of AGE-BSA. After washing the plate with 1X buffer, 50 μl of the standard or the sample are added to wells in duplicate and incubate at room temperature for 10 minutes on orbital microplate shaker. After incubation, 50 μl of diluted anti-AGE antibody were added in each well and incubated at room temperature for 1 hour on orbital shaker. The plate was washed tree time with 250 μl per well of 1X wash buffer and the diluted secondary antibody-HRP conjugate was added at the quantity of 100 μl per well then incubated for 1 hour at room temperature on orbital shaker. After the last washing, the substrate solution was added at the quantity of 100 μl in each well and the reaction was stopped 2–20 mns after with 100 μl of the stop solution. The absorbance was read with the microplate reader at 450 nm as primary wave length and 620 as reference. Concentrations of AGE in samples were calculated with the equation of the standard curve. The test was validated if the coefficient R^2^ was > 0.98.

### 2.4. Determination of plasma sRAGE by ELISA

The quantification of plasma sRAGE was made using the ELISA kit (*RD191116200R*, *sRAGE Human*, *BioVendor)*. The standard, control or sample were added at the quantity of 100μl per well and incubate for 2 hours at room temperature on orbital microplate shaker. The plate was washed 5 times with 350ml of wash solution, 100μl of biotin labelled antibody solution were added into each well and incubate for 1 hour at room temperature on orbital microplate shaker. After a second wash of the plate, 100μl of Streptavidin-HRP conjugate were added into each well then incubated for 30 minutes at room temperature on orbital microplate shaker. After the last washing, the substrate solution was added at the quantity of 100 μl in each well and the reaction was stopped 2–20 minutes after with 100 μl of the stop solution. Absorbance was immediately read with the microplate reader at 450 nm as primary wave length. The quality controls high and low were included in each reaction. Concentrations of AGE in samples were calculated with the equation of the standard curve. The test was validated if the coefficient R2 was > 0.98.

### 2.5. RAGE expression assay by RT-qPCR

#### Total RNA extraction and reverse transcription

The total RNA was extracted from human lymphocytes frozen in nitrogen using *mir*Vana™ *miRNA Isolation Kit (Ref*. *AM1560*, *Ambiom)*. Briefly, after rapid taw of lymphocytes 300–600 μl of the Lysis/Binding Solution were added to disrupt the cells. The cells lysate was vortexed until a homogenous lysate was obtained. The organic extraction consisted to add miRNA Homogenate Additive at 1:10 volume of to the cell lysate, and mixed well by vortexing then left the mixture on ice for 10 min. Acid-Phenol:Chloroform was added to the mixture and vortexed for 1 minute then centrifuged for 5 min at 10,000 x g at room temperature to separate the aqueous and organic phases. The aqueous (upper) phase was transferred into a fresh tube. The 100% ethanol was added at 1.25 volumes to the aqueous phase and 700 μl of the mixture were transferred on to the Filter Cartridge. Centrifugation was made for 15 seconds at 10,000 X g to pass the mixture through the filter. The filter was washed once with 700 μL miRNA Wash Solution 1, then twice with 500 μL of Wash Solution 2/3. RNA was eluted with 100 μl of Elution Solution preheated at 95°C in Nuclease-free tubes and stored at -20°C. The quantity and the quality of RNA at 260/280 and 260/230 were determined by the spectrophotometer *NanoDrop 1000*.

Total RNA was reverse transcribed in to cDNA using the *High-Capacity cDNA Archive Kit (Ref*. *4374966*, *Life Technologies)* according to the manufacturer’s instructions. Reaction was done with 50 μl of 2X RT master mix (containing 10X Reverse Transcription Buffer, 25X dNTPs, 10X random primers, MultiScribe™ Reverse Transcriptase, Nuclease-free H2O) and 50 μL of RNA sample into each wells. Reaction conditions were 25°C for 10 minutes, 37°C for 120 minutes, then stored at -20°C until use.

#### Real-time quantitative PCR assay

The primers used for the PCR were:

Forward—5’CAGGACCCTGGAAGGAAGCAGG3’ and Reverse -5’CTGGTTGTAGAAGAAAGCTTGGC3’. HSPCB was used as reference gene to normalize the PCR. The PCR conditions were as follows: pre-incubation at 95°C for 10 min followed by 40 cycles of 95°C for 15 s, 55°C for 20 s, 72°C for 30, and ending by 72°C for 10 min. PCR was performed with the Light Cycler. The ΔCT method was used to calculate the gene expression.

### 2.6. RAGE gene polymorphism assay

**Fig 1:**
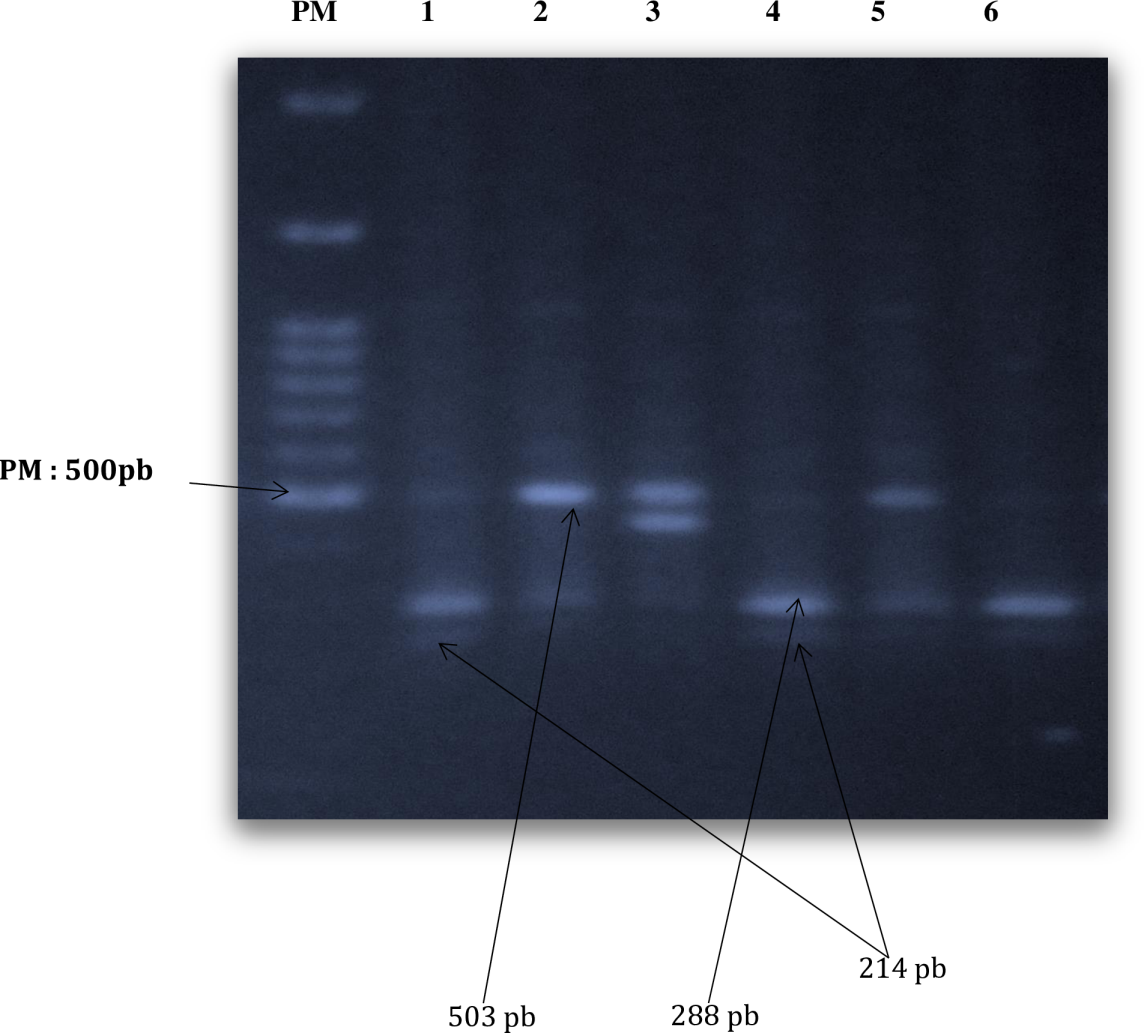
Digestion of PCR product with MfeI showing -374T mutation.

The mutation -374/AT, -429T/C and -69pb deletion/insertion were assessed by RFLP. A fragment of 503pb vas amplified by real-time PCR. The primers used were AGERF 5’- GGG GCA GTT CTC TCC TCA-3’ and AGERR: 5’-TCG TCT TGT CAC AGG GAA TG-3’ as previously described in literature [30]. PCR conditions were 95°C for 10 mn, followed by 35 cycles of 95°C for 30 s, 55°C for 20 s, 72°C for 30 s and a final elongation of 72°C for 10 mns. The PCR products (503 pb) were then digested with MunI (MfeI) and AluI (10μl of PCR product + 1 μl of enzyme + 2 μl of buffer and 18 μl of water) at 37°C for 15 hours in a thermocycler. Digested product (7 μl + 3 μl of loading buffer) were loaded on 1% agar’s gel and migrated for 1 h at 100 volts. Digestion with MfeI revealed fragments 214 and 288 bp for the allele 374A and 503 bp for the mutated allele 374T (Fig 1).

### 2.7. Statistical analysis

Data were analyzed on SPSS, the t-test, ANOVAs (in normal distribution cases), the test for equality of variances (F) and Bonferrony were used for comparison at significance of 0.05. The first step was to determine the prevalence of Plasmodium infection in Fulani and Dogon. We determined the difference of exposure to AGE of Fulani and Dogon by measuring plasma levels of AGE, sRAGE. We then determined the expression of the receptor for AGE (RAGE) and finally we evaluated the relationship between RAGE gene polymorphism and RAGE expression.

## 3. Results

In total 222 volunteers were included in this study. The mean age was 34 years with 3 and 88 years respectively as minimum and maximum. The gender balance was 119/103 (M/F).

**Fig 2:**
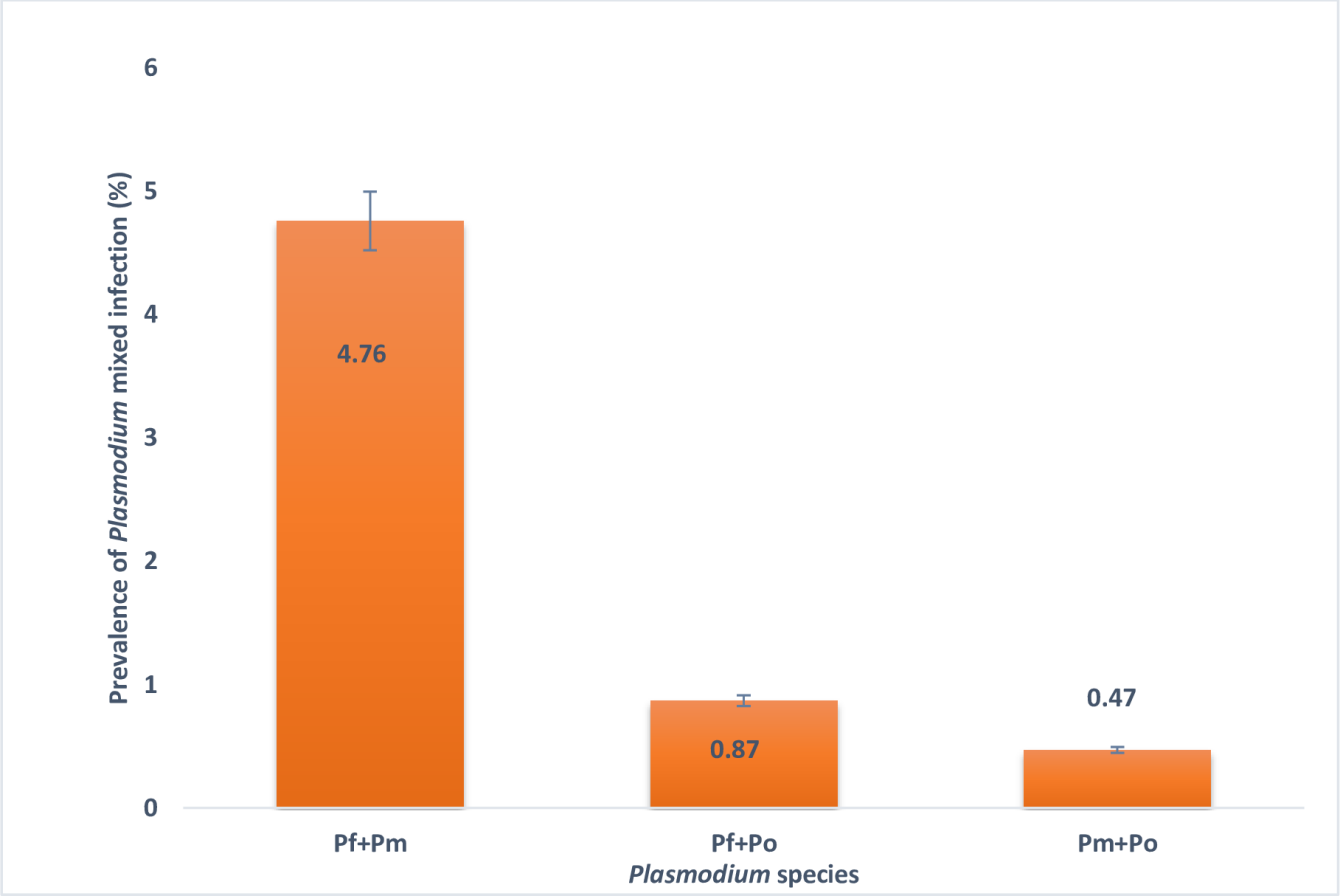
Distribution of mixed infection of Plasmodium in study population.

The prevalence rate of *Plasmodium* in the Fulani and Dogon were respectively 42.64% and 51.30% for *P*. *falciparum* (P>0.05), 5.88% and 6.5% for *P*. *malariae* (P>0.05), 0% and 2.6% for *P*. *ovale* (Table 1), with 6.1% of mixed infection by *Pf+Pm* (4.76%), *Pf+Po* (0.87%) and *Pm+Po* (0.47%) (Fig 2). No case of *P*. *vivax* was formally detected in the study population.

**Table 1:** Molecular prevalence of *Plasmodium* species in Fulani and Dogon.

*Plasmodium* species	Ethnic group		Total
	Fulani	Dogon	
***P*. *falciparum***	29/68 (42.64%)	79/154 (51.30%)	108/222 (48.6%)
***P*. *malariae***	4/68 (5.88%)	10/154 (6.5%)	14/222 (6.3%)
***P*. *ovale***	0	4/154 (2.6%)	4/222 (1.8%)
**Total**	33/68 (48.53%)	93/154 (60.39%)	126/222 (54.95%)

**Fig 3:**
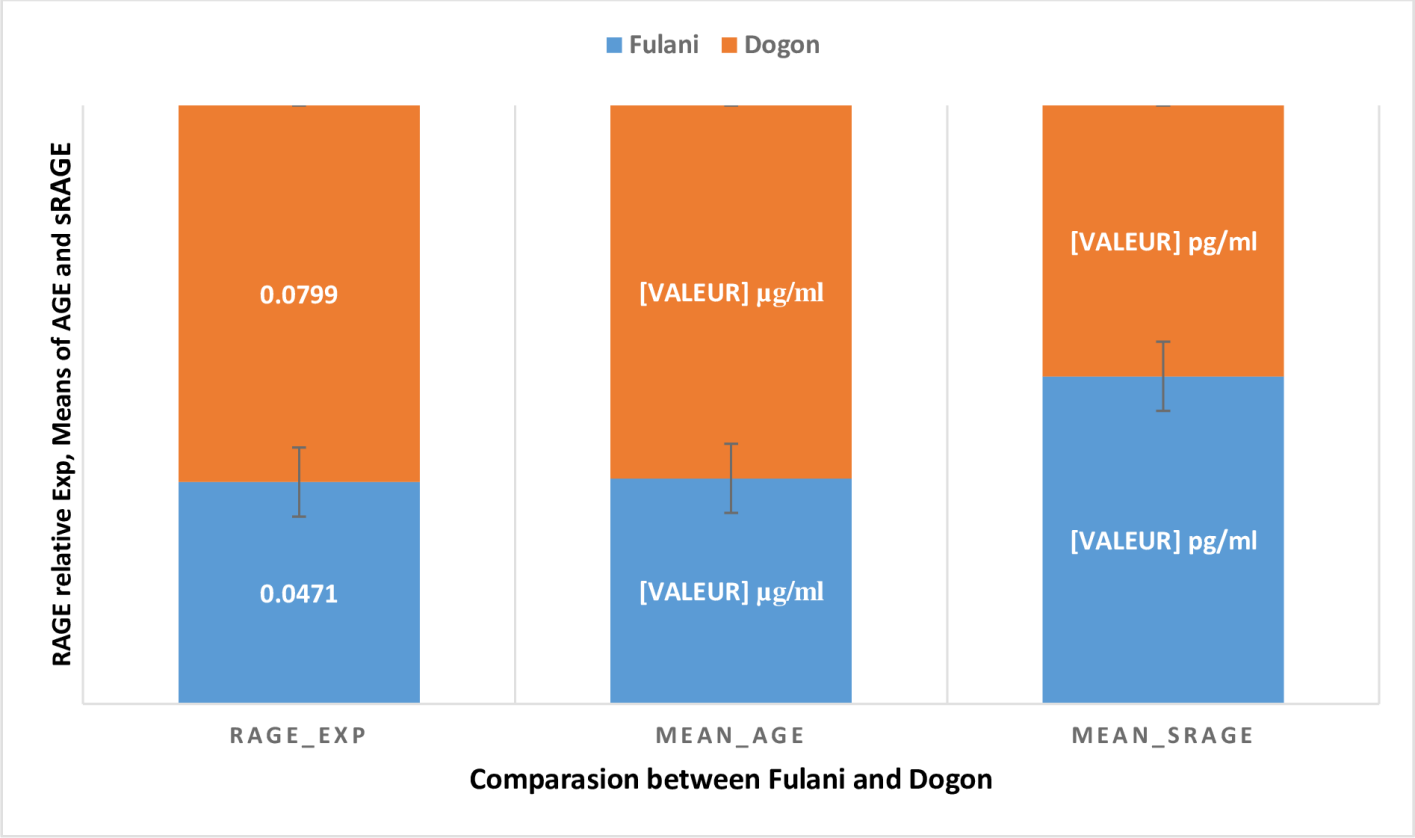
RAGE gene expression, means of AGE and sRAGE in Fulani and Dogon.

The average AGE in the study population was 12.65 μg / ml, and 496.48 pg /ml for sRAGE. In Fulani, the mean of AGE was 10.21μg /μl (95% CI [8.02–10.92]) (Fig 3). The mean of sRAGE receptor was 563,07pg/μl, (95% CI [547.81–580.13]). In Dogon, the mean of AGE was 16.88μg /μl, (95% CI [13.92 to 17.96]) (Fig 3). The mean of sRAGE was 465.68 pg /μl, (95% CI [331.19 to 467.51]).

The Fulani ethic group had the lowest plasma levels of AGE compared to Dogon (10.21 μg/ml +/- 7.81 vs 16.88 μg/ml +/- 11.85, p<0.001) (Fig 3) and the highest levels of sRAGE (563.07 pg/ml vs 465.68 pg/ml, p<0.001) (Fig 3). RAGE was more expressed at two fold in Dogon, compared to Fulani (0.0799 vs 0.0471) but not statistically significant (p = 0.08) (Fig 3).

**Fig 4:**
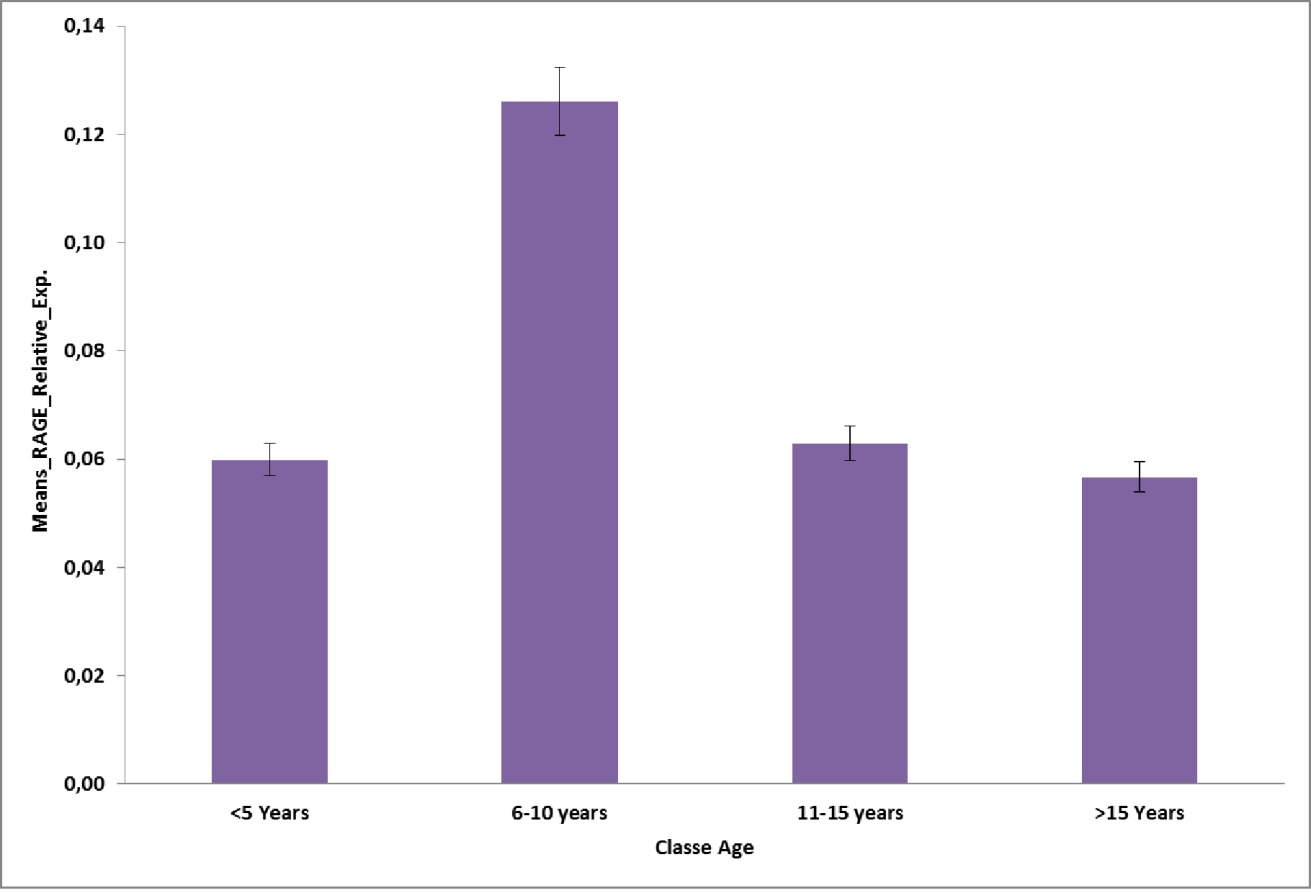
RAGE relative expression by age groups.

Considering the age group of participant, RAGE was more expressed in children at 6–10 years old (Fig 4).

**Fig 5:**
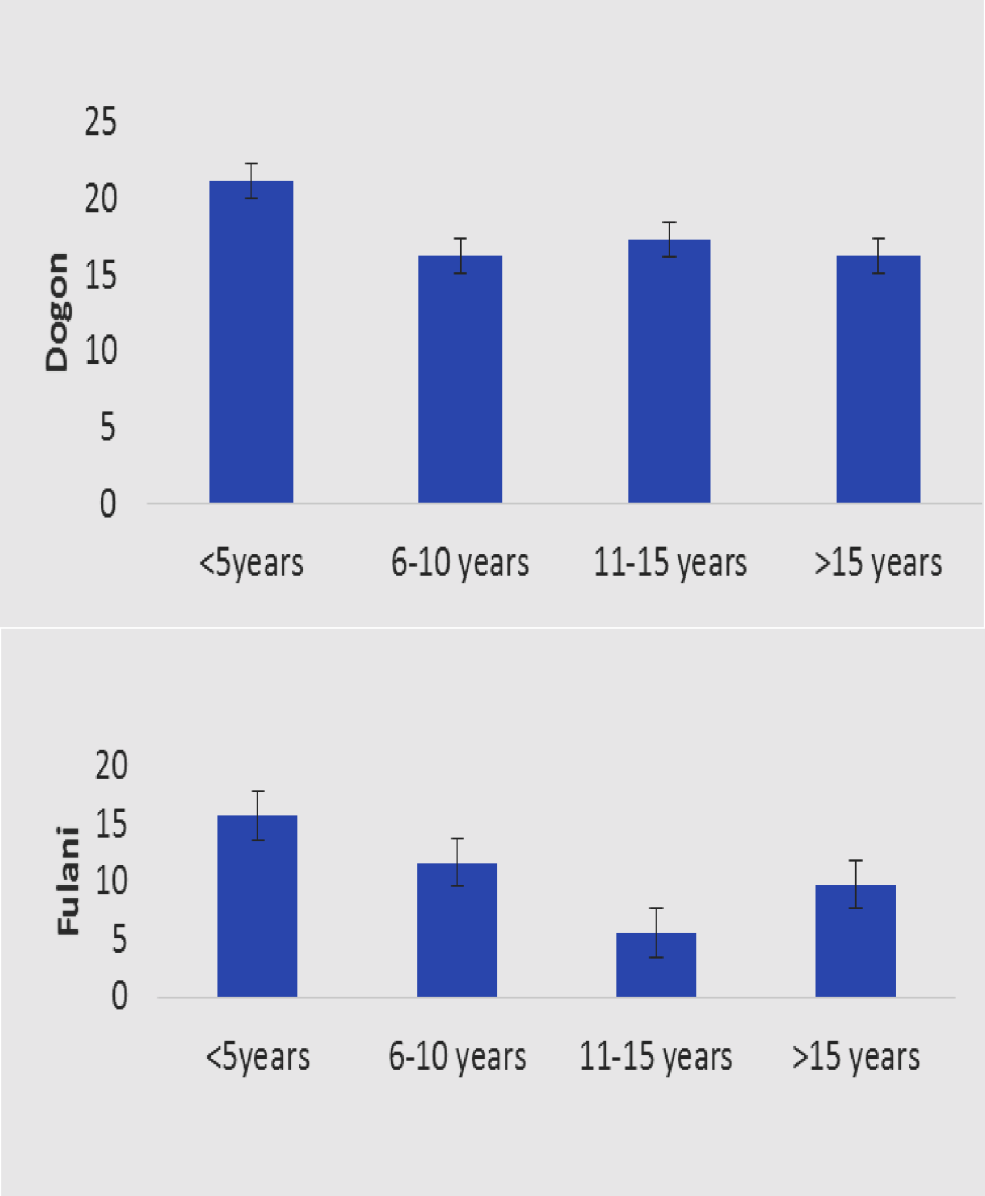
Distribution of the mean of AGE by age groups in Fulani and Dogon.

The mean of AGE was decreasing in Dogon children until the age group of 10 years then remained stable in adulds, while in Fulani it decreased until the age group of 15 years then was increasing in adulds (Fig 5).

**Fig 6:**
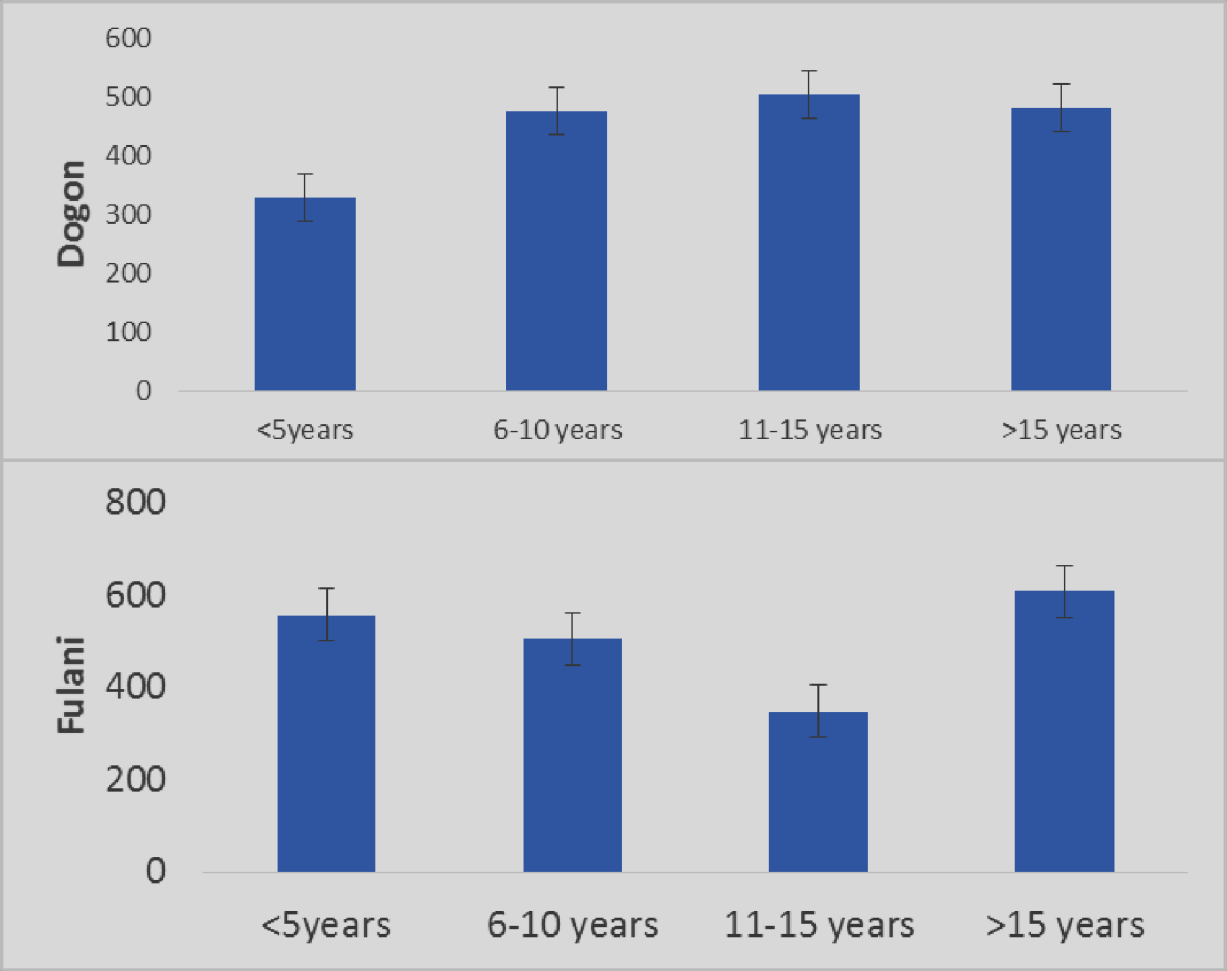
Distribution of the mean of sRAGE by age groups in Fulani and Dogon.

The mean of sRAGE was increasing proportionally with age until 10 years and remained stable in adults of Dogon ethnic group while in Fulani it decreased in children until the age group of 15 years and then increased with age (Fig 6).

High frequencies of the mutated allele -374T were observed in the population (44.28%) with 52.5% in Fulani and 33.3% in Dogon; P<0.01 (Table 2). The -374T mutation was more prevalent in female (58.1%) compared to male (33.3%) (Table 2) and the prevalence of *P*. *falciparum* infection vas high in population without mutation (70% vs 30%) (Table 2). The means of AGE and sRAGE were slightly high in the group without the -374 mutation (Table 2), but not statistically significant.

**Table 2:** Prevalence of -374T mutation by ethnic group, sex and *P*. *falciparum* infection in the study population.

		-374T Mutation		Total
		+	-	
**Ethnic group**	Fulani	52.5% (21)	47.5% (19)	40
	Dogon	33.3% (10)	66.7% (20)	30
**Sex**	Male	33.3% (13)	66.7% (26)	39
	Female	58.1% (18)	41.9% (13)	31
***P*. *falciparum***	+	30% (9)	70% (21)	30
	-	55% (22)	45% (18)	40
**Mean RAGE expression**		0.053 (95% CI [0.029–0.077])	0.064 (95% CI [0.007–0.122])	0.059
**Mean AGE**		9.538	11.587	10.680
**Mean sRAGE**		536.50	567.12	553.57
**Total**		**44.3% (31)**	**55.7% (39)**	**100% (70)**

## 4. Discussions

Samples used in this study have been collected from clinically healthy participants living in malaria endemic area. Samples were collected during the malaria transmission period (from July to September) in cross-sectional surveys. This was a pilot study because none study had yet investigated advanced glycation endproducts in African populations. In this study, we did not assay the other metabolic disorders like diabetes, obesity, as well as the systemic cholesterol and triglyceride levels. This could be a potential limitation of this study.

Two ethno linguistic groups (Fulani and Dogon) living in sympatric have participated to the study. These populations are naturally exposed to the same environmental factors but exhibit different susceptibility to clinical malaria [8,9]. However, the molecular based prevalence of malaria infection determined in our study did not found statistical significant difference between Fulani and Dogon.

The lowest rate of AGE were described in Fulani populations compared to their neighbours Dogon (p<0.001). Fulani and Dogon have different lifestyles, mainly in the diet habits. Fulani are pastoral populations with milk and couscous as main food. Milk and couscous contain very low concentration of AGE [31]. The persistence of the lactase in these populations (>50%) [32] could suggest that they continuously consume milk during their life. The contribution of AGE consumed in food is important in the systemic total AGE [31,33–37]. Exposure to low AGE diet could hence lead to a reduced level of AGE in plasma. The highest plasma levels of AGE were described in Dogon populations. With a diet based mainly on cooked food (at least 3 cooked meals /day), they are more exposed to diet AGE compared to Fulani. The opposite was observed in systemic levels of sRAGE. While Fulani had the higher levels of sRAGE, Dogon exhibited the lowest levels (p<0.001). Several authors have described this soluble form of RAGE as a receptor that plays a decoy role on systemic AGE [38,39]. Our data suggest that the low plasma levels of AGE described in Fulani are due to exposure to a low diet AGE, but also to their capacity to eliminate AGE. However, we did not investigate any functional role of sRAGE in this study.

The overall levels of sRAGE in the study participants were lower compared to these described in non-African populations by other authors [40]. In contrast, the AGE levels were significantly high in Africans. The differences in diet habits could explain these contrasting data. However, the role of other environmental factors related to pollution, infectious diseases should be taken into account.

None differences were observed between age groups or gender for AGE and sRAGE.

The influence of dietary AGE on the expression of RAGE on the immune cells has been described [1]. We investigated the expression of RAGE on lymphocytes in these two populations. RAGE was two-fold more expressed in Dogon compared to Fulani, but there was no statistical difference between the two ethnic groups (p = 0,08). The lack of power of the study could explain the no statistical difference. This study was conducted in non-symptomatic participant. Performing such analysis in patients with clinical malaria could provide better understanding of the expression of RAGE in theses populations. More studies with large sample sizes may be necessary to better investigate the relation between RAGE expression and susceptibility to malaria. However, we did not explore the variants of the RAGE expressed in these populations.

Recruitment of RAGE by AGE leads to a positive feedback loop with auto amplification of RAGE and endogenous generation of AGE, reactive oxygen species (ROS) and advanced oxidation protein end products (AOPP) [2]. This vicious circle leads to chronic oxidative stress and inflammation with impaired innate and adaptive immune responses. The role of diet intake AGE on the innate immunity [1,2] as well as on the adaptive immune response has been established [2]. The presence of RAGE on the immune cells constitutes a link between the environment and immune modulation [1]. Martinez et al., Zoelen et al. have described a positive association between impairment of CD4 T cell response, high plasma levels of AGE and susceptibility of diabetic patients to bacterial infections [27,41]. In experimental animal studies, administration of sRAGE improved survival from sepsis by reducing inflammation and organs injury [39,42]. A positive correlation between plasma level of advanced oxidative proteins products and malaria susceptibility has been also described in children under 2 years [43]. Some polymorphisms of the RAGE promoter gene (374 T/A) are thought to be associated with susceptibility to Human Papilloma virus infection [44], as well as with longevity in elder population. These polymorphisms could be involved in immunity genes expression [4,45].

There are many data supporting ethnic differences in susceptibility to malaria between Fulani and Dogon in Mali [8,9,46]. Enhanced pro inflammatory response has been described in Fulani while in Dogon there was altered innate immune response during malaria infection [9]. The biological factors and mechanisms underlying this difference of immune responses remain not understood. The role of diet in the susceptibility to malaria could be an alternative approach to elucidate the mechanism. Recent papers published have documented the evident role of nutrient-derived metabolites in the development lymphoid organs and the modulation of immune system [3], highlighting the interplay between the immune system and the external environment and specifically the diet. A chronic exposure to diet AGE induces a chronic inflammatory immune response. Repeated stimulation by the same ligand could result in to deregulation of the feedback control [47] and/or exhausted immune cells. Therefore, the inadequate innate immune response observed during malaria infection in Dogon could be a consequence of immune tolerance or degraded immune effectors. The difference of susceptibility to malaria could also be a result of evolutionary process by selecting some mutated allele (374 T) involved in susceptibility/resistance to malaria in these populations. In our study, the mutated allele 374/T of RAGE gene was more frequent in Fulani (52.5%) compared to Dogon (33.3%), (P<0.001). The prevalence of *P*. *falciparum* infection was higher in wild type population compared to the mutated allele population (70% vs 30%, P<0.001).

This mutation has been described as involved in RAGE expression regulation, and could be also involved in the expression of other gene of immunity [48]. In this study, we described a difference but no statistically significant in RAGE expression between wild type and mutated -374T allele population (Table 2). We did not assess the direct implication of theses polymorphisms in the natural resistance to malaria observed in this population, but we suggest more studies in these populations to more investigate a possible association of -374T mutation and malaria susceptibility.

The previous described differences of susceptibility in these populations were related to clinical malaria and spleen enlargement. We did not evaluate the clinical incidence of malaria in this study due to the initial design. But the differences observed in AGE/sRAGE rates and RAGE expression could constitute a good background to design a study to determine the relation between AGE/RAGE and susceptibility to clinical malaria.

The role of PCSK9 mutation (a human cholesterol regulation mutation) has been described in malaria susceptibility. Investigating the role of polymorphism on metabolism regulatory genes could provide valuable information on malaria susceptibility. Multifactor approaches including genetics, immunology, diet, metabolism, and microbiota are also crucial for studies on susceptibility or resistance to malaria in endemic areas.

## 5. Conclusion

Environment factors like diet could play a crucial role in the evolutionary processes leading to the difference of susceptibility/resistance to infectious disease in general and to malaria in particular. A multifactor approach including diet and others environmental and biological factors could provide new insights in our understanding of susceptibility or resistance to malaria in endemic areas. This study is a pilot study that investigated the relation between diet and susceptibility to malaria. There are some limitations for this study due to the none-exploration of other metabolic factors (cholesterol, triglycerides, and diabetes status) and the immune response of PBMCs. In next studies involving malaria clinical cases, these factors could be included to better investigate the relation between AGE-RAGE-immunity and susceptibility to malaria.

## Supporting information

S1 File Data_Base_KT.sav. (SAV)
